# Orthopædic Progress

**Published:** 1905-05-06

**Authors:** 


### ORTHOPÆDIC PROGRESS.

**Tuberculous Arthritis in the Young.**—Robert Jones, of Liverpool, contributes an extremely interesting and valuable paper on this subject. He emphasises its study in the young, because of the clinical differences obtaining in them as contrasted with adults, in whom it runs a much more malignant course, and often requires more heroic measures. Of the symptoms met with in childhood the most important are rigidity and deformity. Rigidity generally precedes pain and is earlier ascertained than muscular shrinkage. In tuberculous arthritis rigidity affects the joint in all directions. In a suspected joint, if there is no rigidity there is no arthritis, and it is more reliable than any other single symptom or group of symptoms. With reference to the hip, he insists on the value of Thomas's flexion test, which is founded upon an inability to extend an inflamed hip without producing lordosis. By lifting the knee of the sound limb until it touches the chest, the pelvis is fixed and the spine is straightened. If there be flexion deformity, the patient is unable to extend the thigh on the diseased side, and it remains at an angle. If the disease be absent, the patient can voluntarily extend his limb. Adverting to the difference between a traumatically and a pathologically dislocated shoulder, Mr. Jones points out that in the former the disability is greatest in the beginning, and as weeks pass by, a fair range of movement results, whereas in the pathological variety, the precise converse obtains and an ankylosed shoulder results. He also strongly advocates rapid correction of the deformity of hip disease, if the child's general condition is satisfactory, and finds that in his experience dissemination of tubercle does not follow more often than when such cases are left alone. His teaching on the treatment of abscess is very sound, and he advocates strongly the value of leaving alone certain types of tuberculous collections so long as possible, that is, if they give rise to no symptoms. He says, "Generally speaking, they are harmless, and are only dangerous if they are infected from without or within. It is a mistake to attach the clinical importance to a tuberculous abscess that we do to a collection of pus. The tuberculous bacillus is not pyogenic; the inflammation it produces runs to granulation tissue. If true suppuration occurs, it is due to a secondary infection."[1] When the skin reddens, then it is time to open the abscess with the minutest aseptic precautions possible, through a half-inch incision, avoiding the use of Volkmann's spoon or irrigator, so as not to interfere with the thick sac wall, which provides so admirable a barrier against infection. Mr. Jones's experience warrants him in saying that hundreds of children die in all parts of England because we do not realise the many responsibilities involved in the routine opening of tuberculous abscesses. As to the test of recovery of a joint, it is cured of disease when the range of movement does not diminish by use; and in those cases resulting in ankylosis, a joint may be pronounced well where the angle does not change after use. Thus, if a patient leaves with a flexion angle of 10° and comes back with one of 15°, recovery has not taken place. The proper pattern and application of Thomas's hip splint are fully discussed, and on these points the original article will well repay perusal, as so many errors are daily in evidence on these matters. Mr. Robert Jones has evolved an original method of overcoming the flexion and adduction deformity of hip disease, the main point being oblique section through the great trochanter, and placing the limb in extension and abduction. Such are a few phases of tuberculous arthritis touched on by the writer of this excellent paper, and we cordially commend its full perusal to those interested in the subject.

[1] Edin. Med. Jour., July 1904, p. 9.

